# Supplementary material for: Chromothripsis during telomere crisis is independent of NHEJ, and consistent with a replicative origin
Source: Genome Res. 2019 May;29(5):737–49. doi: 10.1101/gr.240705.118 (PMC6499312; doi:10.1101/gr.240705.118)
Supplement: Supplemental Material [file supp_gr.240705.118_Supplemental_file_1.zip › contigs/annotated_contigs/DB90/contig.2.DB90_length_456_mean_cov_6.90789473684.docx]

**DB90_length_456_mean_cov_6.90789473684**

```
GTGGTGGCGCACGCCTGTAATCCTAACTACTCAGGTGTCTGAGGCGTGACAATCGCTTGAACCCTAGAAGAGGAGGTGGCAGTGAGGCA
 >chr2:220847597-220847904 - E=2e-170
AAATTCCACACTGCAATCCAGCCTAGGCAACAGAGTGAGACTGTCTAAAACAAAAAATTAAAAAATTTTAAAAAGAATGGCAACAATAG

ATACTGGGGACTAATAGAGTGGGAGTAGGAAGAGGGGCAAGGGCTGAGAAACTAACTACTGGGTACTATGCTCACCACCTGGGTGATGG

GATCGATCATATCAGCATCACACAGTATACCCATGTGAC|A|GGTGCATTATACTTTGGGAGTTGTACTTCTTTAAATAGTCAGCACGG
                                        >chr2:221136525-221136675 - E=1e-75
TAGAGCAGAAAAAGCACTACAGCAGGCGTCAGATGATTGGGTGCTAATCACCGCTTTGCCACTAACTACCTGATGGCCTCAGAGAGGAA

AAATGCTTGCGTT
```
